# Supplementary material for: Trypanosome Infections and Anemia in Cattle Returning from Transhumance in Tsetse-Infested Areas of Cameroon
Source: Microorganisms. 2023 Mar 9;11(3):712. doi: 10.3390/microorganisms11030712 (PMC10058033; doi:10.3390/microorganisms11030712)
Supplement: Supplementary file 1 [file microorganisms-11-00712-s001.zip › microorganisms-2219251-supplementary.pdf]

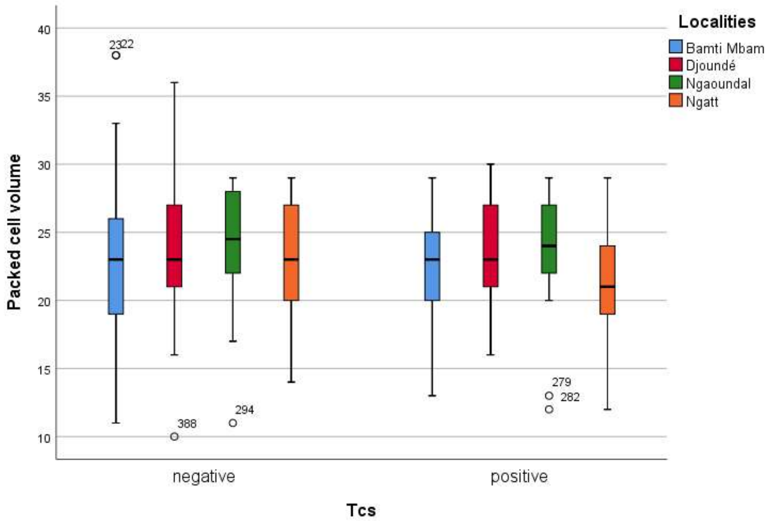

Figure S1. Comparison of haematocrit rates (PCV) of uninfected and of *T. congolense* savanah type infected cattle from the four sampling villages. Tcs infections were identified by PCR.

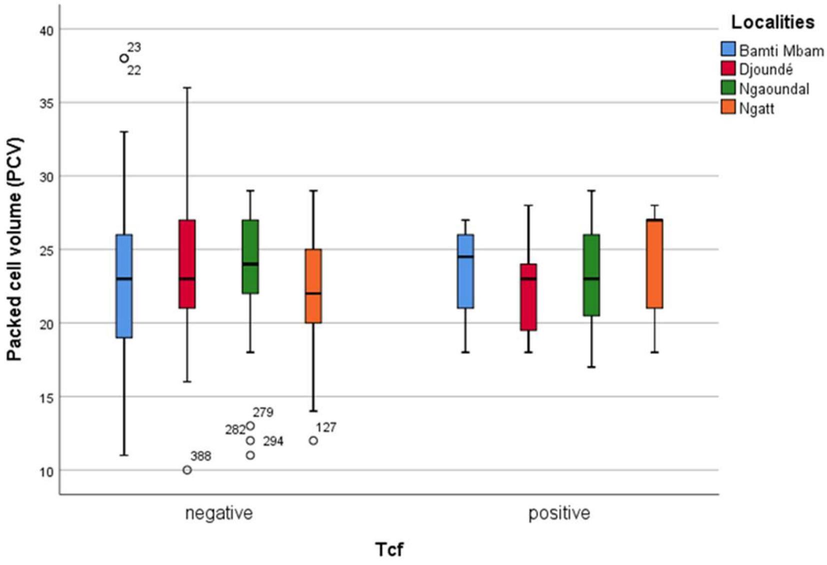

Figure S2. Comparison of haematocrit rates (PCV) of uninfected and of *T. congolense* forest type infected cattle from the four sampling villages. Tcf infections were identified by PCR.

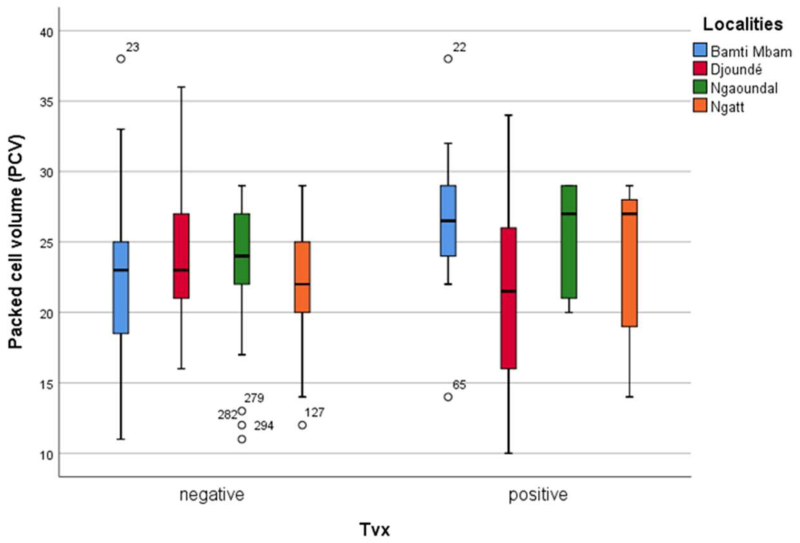

Figure S3. Comparison of haematocrit rates (PCV) of uninfected and of *T. vivax* infected cattle from the four sampling villages. Tvx infections were identified by PCR.

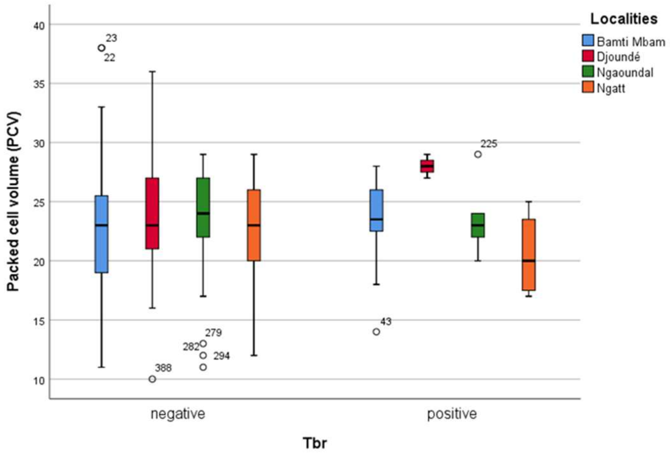

Figure S4. Comparison of haematocrit rates (PCV) of uninfected and of *T. brucei* sl infected cattle from the four sampling villages. Tcs infections were identified by PCR.

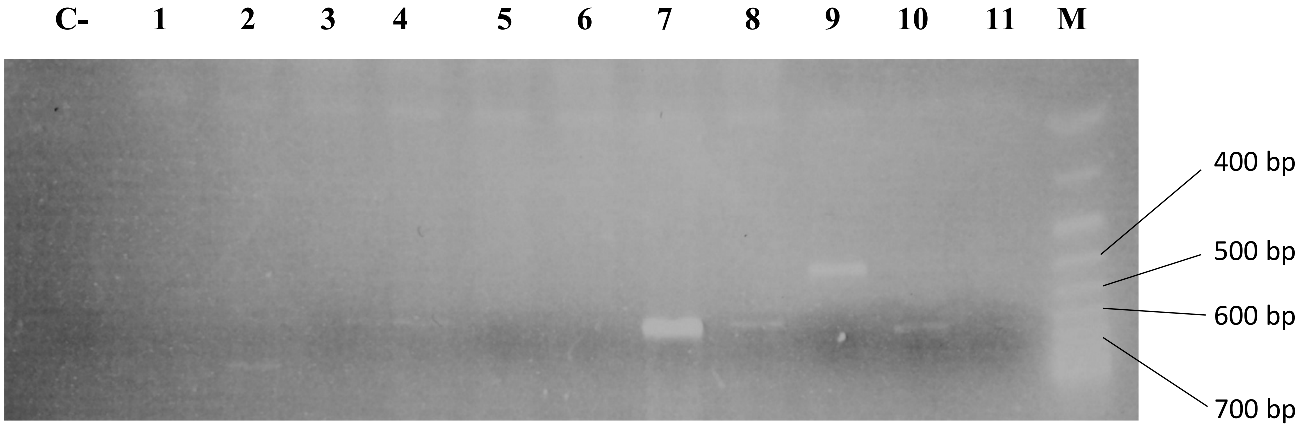

Figure S5: 2% agarose gel showing the size of the trypanosomes bands.

2 –non identified band

4,8-*Trypanosoma congolense* Savannah type (697 bp)

7,10 – *Trypanosoma congolense* forest type (714 pb)

9- *Trypanosma brucei* (480 pb)

1,3,5,6,11- negative samples

M-marqueur de taille de poids moléculaire 100

800 pb
700 pb
600 pb
500 pb
